# The cerebral metabolic topography of spinocerebellar ataxia type 3

**DOI:** 10.1016/j.nicl.2018.03.038

**Published:** 2018-03-29

**Authors:** Sanne K. Meles, Jelmer G. Kok, Bauke M. De Jong, Remco J. Renken, Jeroen J. de Vries, Jacoba M. Spikman, Aaltje L. Ziengs, Antoon T.M. Willemsen, Harm J. van der Horn, Klaus L. Leenders, Hubertus P.H. Kremer

**Affiliations:** a Department of Neurology, University of Groningen, University Medical Center Groningen, The Netherlands; b Neuroimaging Center, Department of Neuroscience, University of Groningen, University Medical Center Groningen, The Netherlands; c Department of Neuropsychology, University of Groningen, University Medical Center Groningen, Groningen, The Netherlands; d Department of Nuclear Medicine and Molecular Imaging, University of Groningen, University Medical Center Groningen, The Netherlands

**Keywords:** Spinocerebellar ataxia type 3, Neuroimaging, Cerebellum, FDG-PET, Networks

## Abstract

**Introduction:**

We aimed to uncover the pattern of network-level changes in neuronal function in Spinocerebellar ataxia type 3 (SCA3).

**Methods:**

17 genetically-confirmed SCA3 patients and 16 controls underwent structural MRI and static resting-state [^18^F]‑Fluoro‑deoxyglucose Positron Emission Tomography (FDG-PET) imaging. A SCA3-related pattern (SCA3-RP) was identified using a multivariate method (scaled subprofile model and principal component analysis (SSM PCA)). Participants were evaluated with the Scale for Assessment and Rating of Ataxia (SARA) and with neuropsychological examination including tests for language, executive dysfunction, memory, and information processing speed. The relationships between SCA3-RP expression and clinical scores were explored. Voxel based morphology (VBM) was applied on MRI-T1 images to assess possible correlations between FDG reduction and grey matter atrophy.

**Results:**

The SCA3-RP disclosed relative hypometabolism of the cerebellum, caudate nucleus and posterior parietal cortex, and relatively increased metabolism in somatosensory areas and the limbic system. This topography, which was not explained by regional atrophy, correlated significantly with ataxia (SARA) scores (ρ = 0.72; *P* = 0.001). SCA3 patients showed significant deficits in executive function and information processing speed, but only letter fluency correlated with SCA3-RP expression (ρ = 0.51; *P* = 0.04, uncorrected for multiple comparisons).

**Conclusion:**

The SCA3 metabolic profile reflects network-level alterations which are primarily associated with the motor features of the disease. Striatum decreases additional to cerebellar hypometabolism underscores an intrinsic extrapyramidal involvement in SCA3. Cerebellar-posterior parietal hypometabolism together with anterior parietal (sensory) cortex hypermetabolism may reflect a shift from impaired feedforward to compensatory feedback processing in higher-order motor control. The demonstrated SCA3-RP provides basic insight in cerebral network changes in this disease.

## Highlights

• A metabolic cerebral pattern could be identified in FDG-PET data of SCA3 patients, which was not explained by regional atrophy.
• Striatum decreases in the SCA3-pattern reflect extrapyramidal involvement.
• The SCA3-pattern reflects changes in higher-order motor control.

## 1 Introduction

Spinocerebellar ataxia type 3 (SCA3, previously coined Machado-Joseph disease) is a neurodegenerative disease caused by trinucleotide (CAG) repeat expansion in exon 10 of the ATXN3 gene on chromosome 14 (p32). The cerebellum is most severely affected, with ataxia as key feature, but patients may also develop pyramidal and extra-pyramidal signs, neuropathy and oculomotor dysfunction (Riess et al., 2008), as well as cognitive problems (Braga-Neto et al., 2014).

Pathology in variable brain structures (including the cerebellum, brainstem, thalamus, basal ganglia, and cerebral cortex) (Seidel et al., 2012; Rub et al., 2008; Rub et al., 2013) may explain the variety of symptoms, although dysfunction may also arise from functional disconnection from the cerebellum. Imaging cerebral metabolism with [^18^F]‑Fluoro‑deoxyglucose Positron Emission Tomography (FDG-PET) enables assessment of distributed cerebral dysfunction (Reivich et al., 1979). The obtained FDG-PET (as well as perfusion SPECT) data are commonly analyzed with univariate models which have consistently demonstrated cerebellar hypo-activity, but showed variable results regarding the involvement of extra-cerebellar structures such as the brainstem (Soong et al., 1997; Soong and Liu, 1998; Wullner et al., 2005; Wang et al., 2007), thalamus (Wullner et al., 2005), lentiform nucleus (Wang et al., 2007), limbic lobe (Wang et al., 2007; Braga-Neto et al., 2012a), and occipital cortex (Soong et al., 1997; Soong and Liu, 1998; Braga-Neto et al., 2012a).

While univariate approaches treat every region (voxel) individually, multivariate analysis enables assessment of network-level alterations. With the scaled subprofile model with principal component analysis (SSM PCA) (Spetsieris and Eidelberg, 2011) disease-specific patterns have been identified in several neurodegenerative disorders (Meles et al., 2017; Niethammer and Eidelberg, 2012).

Here, we studied FDG-PET data of SCA3 patients with SSM PCA to gain insight into the pattern of changed neuronal activity in this disease. To understand the relation with symptoms, correlations between expression of the SCA3 metabolic pattern and clinical parameters were analyzed. Moreover, to assess whether the metabolic pattern reflects network-level activity changes beyond regional atrophy due to neuronal cell-loss, we additionally performed a voxel-based morphometry (VBM) analysis on structural MRI data to compare FDG uptake with grey matter loss.

## 2 Methods

### 2.1 Participants

We included 17 genetically-confirmed SCA3 patients. Inclusion criteria were age 18–65 years and absence of other neurological disorders. In addition, we studied 16 age, gender and education-matched healthy controls. Healthy controls did not have a history of neurological disease, nor a family history of cerebellar disorders. Severity of ataxia was assessed in both groups by an experienced neurologist (HPHK, JJdV) with the Scale for Assessment and Rating of Ataxia (SARA) (Schmitz-Hubsch et al., 2006).

Neuropsychological tests selected for this study were based on previously published results in SCA3 patients (Braga-Neto et al., 2014). These tests evaluated language (Semantic Fluency), memory (Dutch version of the Rey Auditory Verbal Learning Test; RAVLT), and executive (Letter Fluency) domains. Information processing speed was measured with the Symbol Digits Modalities test (SDMT). Affective symptoms were assessed with the Hospital Anxiety and Depression Scale (HADS).

The study was approved by the Ethics Committee of the University Medical Center Groningen (NL45036.042.13). Voluntary written informed consent was obtained from each subject after verbal and written explanation of the study, in accordance with the Declaration of Helsinki.

### 2.2 Image acquisition

Static FDG-PET scanning was performed on a Siemens Biograph mCT-64 PET/CT camera (Siemens, Munich, Germany) in a three-dimensional mode, 30 min after intravenous injection of 200 MBq of ^18^F-FDG, with a frame-duration of 5 min. A low-dose computed tomography transmission scan was performed for attenuation correction. Images were reconstructed with OSEM3D, including point-spread function and time-of-flight modeling (3 iterations/21 subsets, matrix 400) and smoothed with a Gaussian 2 mm full-width at half-maximum filter. Central nervous system depressants were discontinued in all subjects for at least 24 h before FDG-PET scanning. This included clonazepam, which was used by three patients. One patient used levodopa at the time of the study, which was not discontinued. FDG uptake and image acquisition were performed in a resting state with the subject's eyes closed in a dimly lit room with minimal auditory stimulation.

MRI-T1 images were acquired for registration purposes. Subjects were scanned at a Philips Achieva 3.0T MRI scanner (Philips, Best, The Netherlands) with a 32-channel head coil. A 3D T1 TFE image was acquired for each subject using the following parameters: 160 sagittal slices without gap, FOV (ap × rl × fh) 256 × 160 × 256 mm, acquired matrix 256 × 256, voxel size 1 × 1 × 1 mm, repetition time 7.8 ms, echo time 3.6 ms and flip angle 8 degrees for a total scan duration of 10 min and 14 s.

### 2.3 Image registration

Prior to the SSM PCA analysis, the PET images of all subjects needed to be in registration. First, from the T1 images the brains were extracted from the rest of the head using FreeSurfer (Segonne et al., 2004; FreeSurfer, 2012) (version 5.3, with default parameters, running on Ubuntu 12.04.5 LTS), resulting in a brain mask per subject. Tools from the FSL (Smith et al., 2004) toolbox (version 5.0.8, running under Ubuntu 10.04 LTS) were used for subsequent registration. Using FLIRT (Jenkinson and Smith, 2001; Jenkinson et al., 2002), the PET images were registered to the brain mask produced by FreeSurfer (i.e. intra-subject registration), and the transformation parameters were stored. Here, default parameters were used except for the cost and search-cost options (set to normalized mutual information) and the degrees of freedom (6, i.e. allowing for rotations and translations). The MRI data were registered to the MNI 2 mm template provided with FSL by first using FLIRT to register the subject's brain mask linearly with the MNI 2 mm brain image (using default parameters). The resulting transformation parameters were used as a starting estimate for the nonlinear registration of the subject's T1 image with the MNI 2 mm head volume using FNIRT (Andersson et al., 2007) (using default parameters, as stored in the T1_2_MNI152_2mm.cnf file supplied with FSL). The linear transformation parameters resulting from registering the PET image to the brain mask and the nonlinear transformation parameters resulting from registering the T1 image to the MNI volume were combined (to minimize the number of interpolation steps) and applied to the PET images. Finally, these images were smoothed with an 8 mm full-width at half-maximum filter Gaussian kernel and stored for subsequent analyses.

### 2.4 Identification of the SCA3-RP

We applied an automated algorithm written in-house, based on the SSM PCA method of Spetsieris et al. (2015), implemented in Matlab (version 2012b; MathWorks, Natick, MA). First, a 35% threshold of the whole-brain intensity maximum was applied to each individual FDG-PET image to remove out-of-brain voxels; these were multiplicatively combined to create one common mask that included only non-zero values for all subjects. This mask was applied to all images. Masked images were log-transformed and subject mean and group mean were removed, resulting in a Subject Residual Profile per subject. Principal component analysis (PCA) was applied in voxel space, and the principal components explaining the top 50% of the total variance were selected for further analysis.

For each subject, a score was calculated for each principal component, by projecting the Subject Residual Profile on each principal component. Components that gave maximum discrimination between controls and SCA 3 patients (based on these scores) were identified with a stepwise logistic regression procedure, using the lowest Akaike information criterion of the model as a selection criterion. If more than one component was identified, then these components were linearly combined to form one pattern. Each component in this pattern was weighted by the coefficient obtained from the logistic regression model. The final pattern was termed the SCA3 related pattern (SCA3-RP).

### 2.5 Validation of the SCA3-RP

A previously identified pattern can be used to quantify the FDG-PET scans of new subjects. In this procedure, an individual's scan is projected onto the pattern, resulting in a single score (Spetsieris et al., 2015). These pattern expression scores can be determined in a new cohort of patients and controls (a testing set) for validation of the pattern. A disease-related pattern is considered valid if a significant difference in pattern expression scores can be demonstrated between patients and controls in the testing set. After such validation, the pattern may be applied to new images.

In this study, a testing sample was not available. Therefore, we performed a leave-one-out cross validation (LOOCV) on our identification cohort. With LOOCV, SCA3-RP identification (i.e. SSM PCA) was repeated 33 times, each time leaving out one subject. For example, patient 1 is left out, and the pattern is re-determined on the remaining 16 patients and 16 controls. Subsequently, the subject score of this pattern is calculated for patient 1. This procedure is then repeated for each subject, thus generating expression z-scores for each subject (controls and patients), independent from the pattern identification step. The difference in LOOCV z-scores between controls and patients was tested for significance with a student's *t*-test. If significant, we consider the pattern a good predictor of SCA3 in new cases. LOOCV hereby enables an estimation of diagnostic performance in just one dataset (Arlot and Celisse, 2010). This approach was also applied in previous FDG PET SSM PCA studies (Teune et al., 2013)

To determine sensitivity and specificity of the SCA3-RP, a receiver operating curve (ROC) was plotted based on the original SCA3-RP z-scores. The cut-off z-score that gave optimum sensitivity and specificity (based on the ROC inflection point) in the identification cohort was chosen as the threshold. This threshold was subsequently applied to the LOOCV z-scores in controls and patients, and sensitivity and specificity of the SCA3-RP was determined.

An important issue is that pattern maps (voxel weights and signs) can fluctuate depending on which sample of controls and patients was used for pattern identification. Although this variability does not hamper diagnostic ability (Habeck and Stern, 2010), it is important to determine which regions in the pattern are stable and can be interpreted as part of the pathophysiological process. A statistical approach to solve this is a bootstrap procedure. This entails repeating the pattern identification process (SSM PCA and subsequent logistic regression) multiple times on randomly sampled data with replacement. This yields multiple slightly different patterns and thus a distribution of weights per voxel. Using this distribution, confidence intervals per voxel can be determined. Voxels with confidence intervals straddling zero can be interpreted as non-informative and are therefore excluded from the visualization. Here, we performed 1000 repetitions, and applied a one-sided confidence interval threshold of 95% (percentile method) followed by visualization of the stable voxels.

SSM PCA provides a mathematical decomposition of components that do not necessarily each have intrinsic biological meaning. The biological meaning of a pattern is supported when pattern expression values show a significant relationship with other markers of disease. Therefore, we tested the correlation between SCA3-RP LOOCV z-scores and SARA (sub)scores. To investigate whether the SCA3-RP topography may explain neuropsychological deficits in SCA3 patients, we explored correlations between SCA3-RP LOOCV z-scores and the tests from the neuropsychology evaluation.

### 2.6 Statistical analysis

Between-group differences in age and SCA3-RP z-scores were assessed using the student's *t*-test. Differences in SARA scores and neuropsychology test-scores were assessed using the Mann-Whitney *U* test. In SCA3 patients, CAG repeat length was correlated to age and age at onset using a Spearman correlation coefficient. SCA3-RP LOOCV z-scores were correlated to age and age at onset using Pearson's r correlation coefficient. SCA3-RP LOOCV z-scores were correlated to SARA (sub)scores, disease duration, CAG repeat length, tests in the language, executive and memory domains, information processing speed, and affective symptoms using a Spearman's correlation coefficient. To correct for multiple comparisons, we considered results to be significant if *p* < 0.005 (=0.05/10). All analyses were performed using SPSS software version 23 (SPSS Inc., Chicago, IL).

### 2.7 Structural MRI analysis

To investigate differences in grey matter atrophy, a voxel based morphometry (VBM) analysis was conducted using SPM12 (Ashburner and Friston, 2000). The T1-weighted MR images were first reoriented manually to ensure that the origin was close to the anterior commissure. Subsequently, images were segmented into grey matter, white matter, and cerebrospinal fluid. Grey matter segmentations were spatially normalized to MNI-space using the Diffeomorphic Anatomical Registration using Exponentiated Lie algebra (DARTEL), and smoothed with a 10 mm full width at half maximum kernel (Ashburner, 2007). A two sample *t*-test was performed to analyze the difference in brain atrophy between patients and healthy controls. To correct for inter-individual differences in total grey matter volume, data were scaled to the total grey matter volume per subject (calculated using the Tissue Volumes function in spm12). Resulting clusters were considered statistically significant if they survived an FWE corrected cluster *p* value of 0.05 after applying an initial cluster defining threshold of *P* < 0.001.

Additionally, a region of interest (ROI) analysis was performed to investigate possible correlations between local grey matter atrophy and FDG uptake in specific ROIs. ROIS from the Automated Anatomical Labeling (AAL) atlas (in MNI space) (Tzourio-Mazoyer et al., 2002) were selected based on the regions in the SCA3-RP and previous VBM studies (Hernandez-Castillo et al., 2017) in SCA3. ROIs included the brainstem, cerebellum (hemispheres and vermis), the caudate nucleus, putamen, pallidum, occipital lobe, postcentral gyrus, and superior frontal gyrus.

Binary masks were made for each ROI. Subsequently, these masks were applied to the individual grey matter segmentations and FDG images using spm12, yielding total grey matter volume and FDG uptake for each ROI in every subject. The total FDG uptake in every ROI was normalized to the total brain uptake of each individual. In case of lateralized ROIs, the sum of grey matter volume and FDG uptake of left and right was used for further analyses. As regional FDG uptake and grey matter volume were normally distributed, Pearson correlations were calculated between grey matter volume and FDG uptake in every ROI using SPSS. Correlations between SARA scores, disease duration, grey matter volume and FDG uptake in each ROI were explored with Spearman's rho. Correlations were considered significant at *P* < 0.05 (uncorrected).

## 3 Results

### 3.1 Participants

Clinical features of the studied cohort are presented in Table 1. As expected, higher SARA scores characterized the SCA3 patients. They further showed significantly lower scores on letter fluency and the SDMT compared to controls. In addition, SCA3 patients had significantly higher scores on the HADS depression scale.

**Table 1:** Clinical features of the studied cohort.

	Healthy controls (*n* = 16)	SCA 3 patients (*n* = 17)	*P*-value^b^	Correlation with SCA3-RP LOOCV z-scores in SCA3 patients (coefficients and *p*-values)
Age, years (range)	49.4 ± 13.5 (20–65)	45.3 ± 11.4 (24–62)	0.36	*r* = 0.03; *P* = 0.91
n Male (%)	8 (50.0)	9 (53.9)	0.87	
Age at onset, years (range)		35.6 ± 10.2 (20–55)		r = 0.03; P = 0.91
Disease duration (range)		9.7 ± 7 (3−30)		*r* = 0.05; *P* = 0.85
CAG repeats^a^ (range)		70 (65.5–72.5)		ρ = 0.35; *P* = 0.16
SARA^a^ (range)	0 (0–0.5)	10 (3.5–15.5)	<0.001⁎	ρ = 0.75; *P* = 0.001⁎
Level of education^a^	5.50 (5–6)	5.0 (5–6)	0.31	ρ = −0.28; *P* = 0.28
Anxiety (HADS)	4.0 ± 2.4	3.5 ± 2.9	0.63	ρ = −0.27; *P* = 0.29
Depression (HADS)	1.6 ± 1.5	5.3 ± 3.5	0.001⁎	ρ = 0.03; *P* = 0.90
Semantic Fluency	26.2 ± 7.8	20.0 ± 6.5	0.028	ρ = −0.19; *P* = 0.46
Letter Fluency	38.6 ± 11.5	24.3 ± 12.0	0.001⁎	ρ = −0.51; P = 0.04
Immediate Recall (RAVLT)	50.7 ± 9.0	42.6 ± 9.5	0.021	ρ = −0.19; *P* = 0.48
Delayed Recall (RAVLT)	11.4 ± 3.0	8.8 ± 2.8	0.010	ρ = −0.07; *P* = 0.80
Symbol Digit Modalities Test	62.5 ± 9.6	44.4 ± 8.2	<0.001⁎	ρ = −0.24; *P* = 0.36

Values are given as mean ± SD unless otherwise specified.

a Median (interquartile range).

b Independent samples t-test for age, Chi^2^ for gender, Mann-Whitney U test for remaining variables.

⁎ Significant at *P* < 0.005; RAVLT = Rey Auditory Verbal Learning Test; r = Pearson's r correlation coefficient for parametric variables; ρ = Spearman's rho correlation coefficient for non-parametric variables.

Patients with longer CAG repeat lengths had an earlier onset of symptoms (ρ = −0.78, *P* < 0.001, Supplementary Fig. 1). CAG repeat length was also significantly and inversely correlated with age (ρ = −0.82, *P* < 0.001). This merely reflects homogeneity of the studied cohort in terms of clinical presentation. Older patients had lower CAG repeat lengths and similar clinical severity as younger patients with more expanded alleles.

### 3.2 Identification of the SCA3-RP

After applying SSM PCA, the first seven principal components explained 50.2% of the total variance, and were used for further analysis. A weighted linear combination of components 1 and 5 (relative weights: 1 and 2.5, respectively; variance explained: 17.0% and 5% respectively) could best discriminate between controls and patients in the logistic regression model. The (weighted) linear combination of components 1 and 5 was termed the SCA3-RP.

**Fig. 1:**
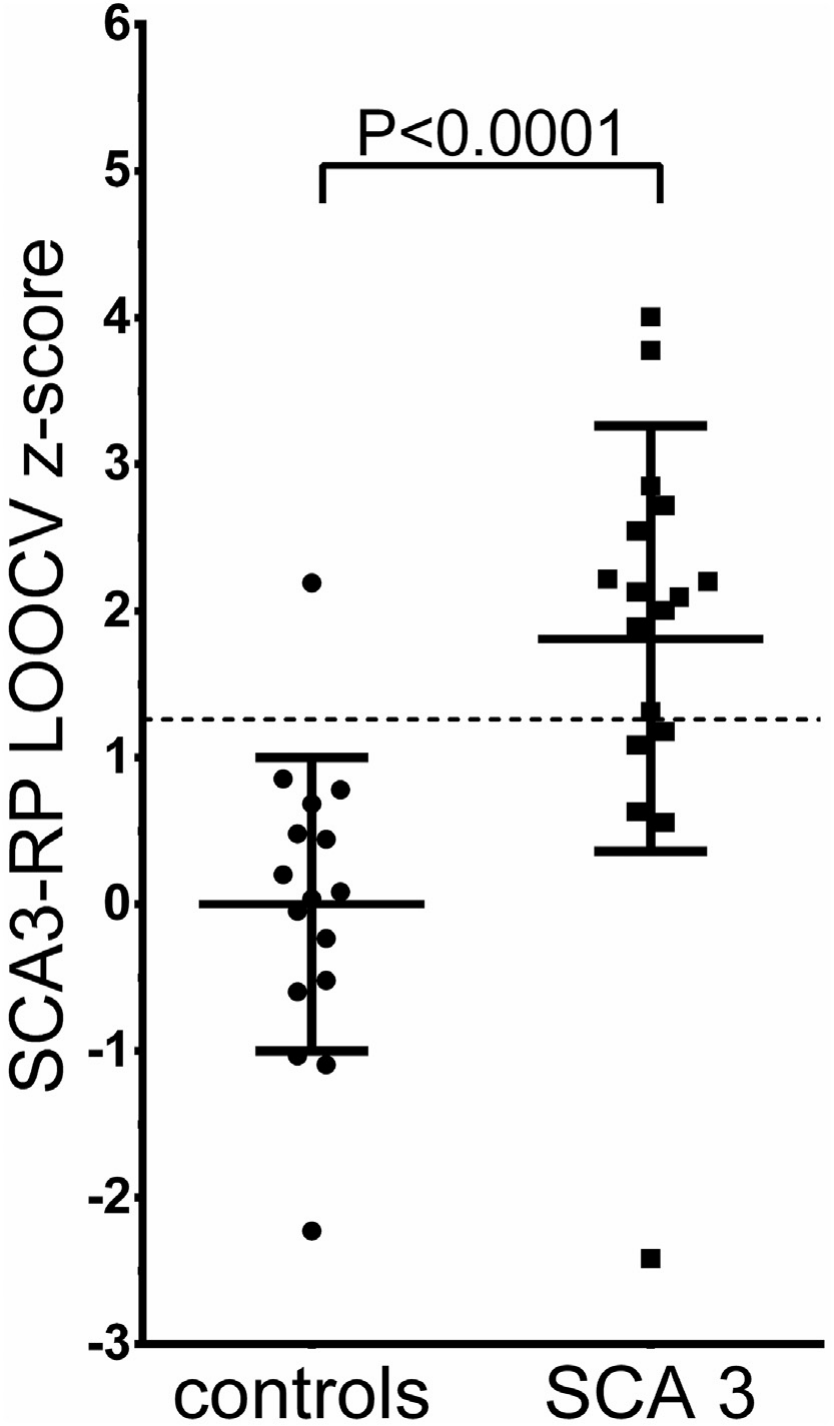
Group differences in SCA3-RP expression z-scores SCA3-RP expression z-scores were validated with a leave-one-out cross validation (LOOCV) and plotted for controls and SCA3 patients. The line at y = 1.26 indicates the threshold, determined on the original SCA3-RP z-scores. Bars indicate mean and standard deviation.

SCA3-RP subject z-scores for each subject were entered in a ROC analysis. The optimum cut-off z-score was determined at z = 1.26. For validation, SCA3-RP LOOCV z-scores were obtained. The threshold (z = 1.26) was applied to the validated scores, which gave a sensitivity of 70.6% and a specificity of 93.8%. SCA3-RP LOOCV z-scores are plotted in Fig. 1, which shows a significant difference between groups (*P* < 0.0001).

### 3.3 Topography of the SCA3-RP

**Fig. 2:**
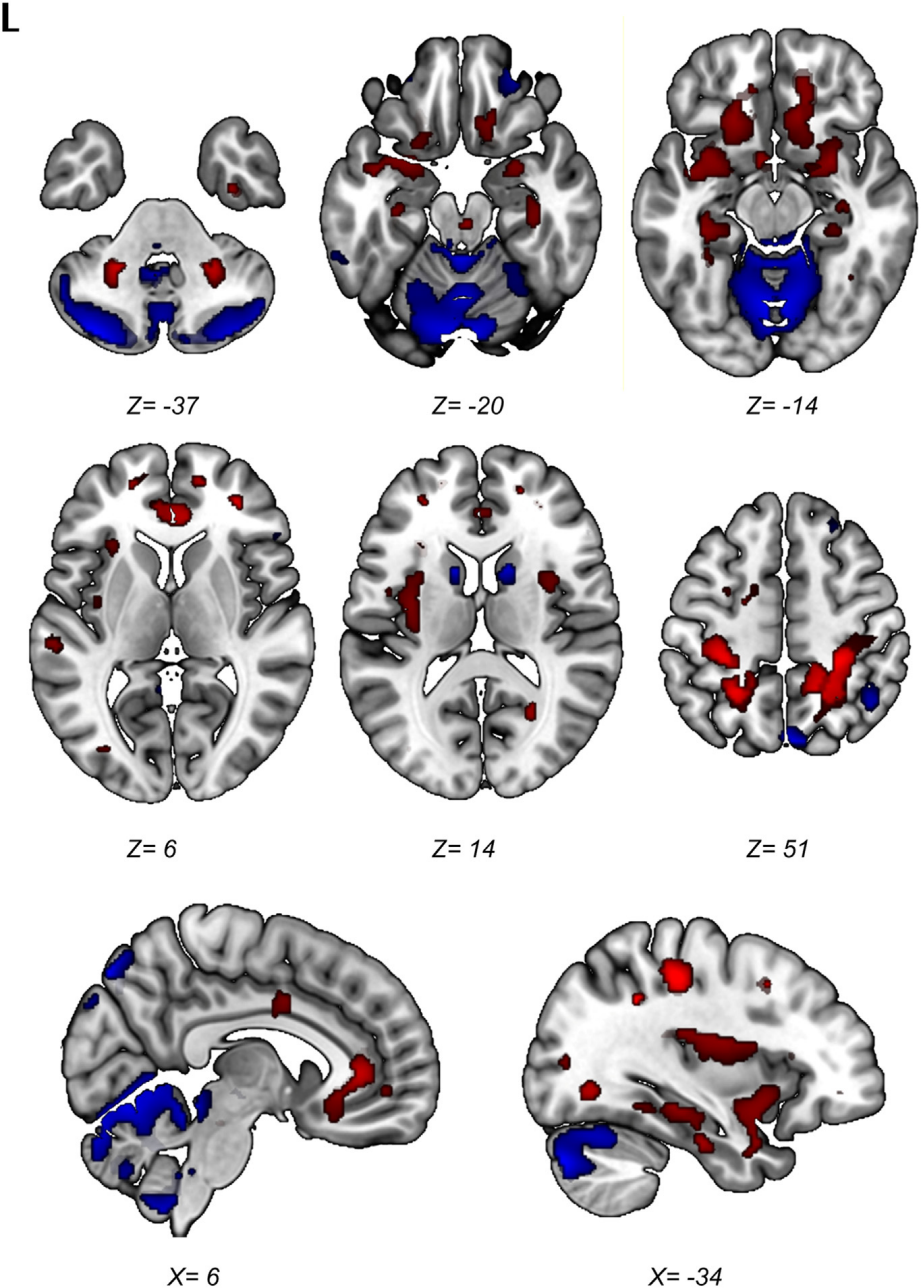
Topography of the SCA3-RP. Stable voxels of the SCA3-RP on a T1 MRI template. Red = relative hypermetabolism; Blue = relative hypometabolism. Color intensity reflects the median of the values resulting from the bootstrap resampling. L = left. Coordinates are in Montreal Neurological Institute (MNI) standard space.

Regions that survived the 95% confidence interval after bootstrap resampling are displayed in Fig. 2. This visualization was used to draw conclusions about the most important (and reliable) regions likely to be involved in SCA3. Stable and consistent relative hypometabolic regions included the cerebellum and cerebellar vermis, the lower brainstem and midbrain (tectum), the caudate nucleus, and small clusters in the posterior parietal cortex (left and right precuneus [BA 7] and the right inferior parietal lobule [BA 39]). Symmetric relative metabolic increases were observed in the cerebellar peduncles, the amygdala, hippocampus, parahippocampal gyrus, the orbitofrontal cortex (BA 11), insula, the anterior and middle cingulate gyrus and the somatosensory areas in the parietal cortex (BA 3,2 and 5).

When all, unthresholded voxels in the SCA3-RP are considered (Supplementary Fig. 2), a highly symmetrical topography is revealed with lower voxel weights in the cerebellum, striatum and thalamus, lateral prefrontal cortex, primary motor cortex, medial and posterior parietal cortex, and the occipital cortex. A visual representation of the average relative FDG distribution per group (patients and controls separately) is provided in Supplementary Fig. 3.

### 3.4 Correlation between SCA3-RP expression z-scores and clinical metrics

**Fig. 3:**
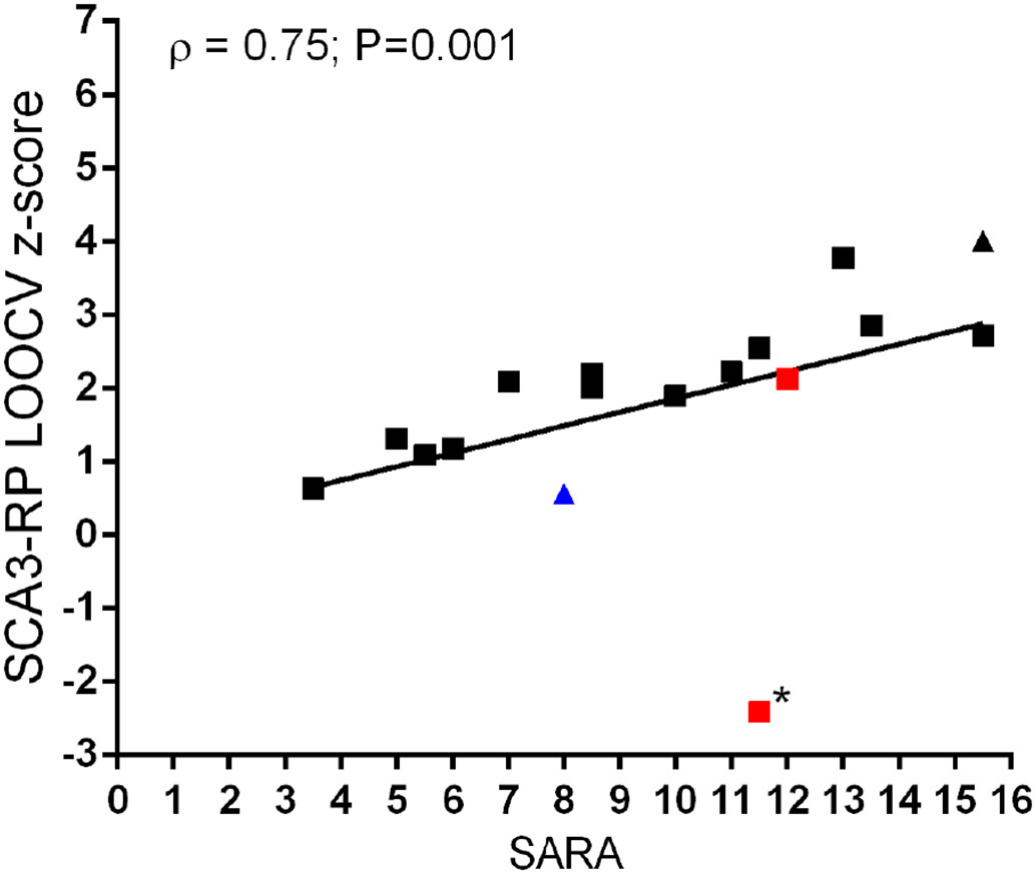
Correlation between SCA3-RP LOOCV z-scores and SARA scores. Two subjects had parkinsonism (indicated in red), and one had dystonic features (blue), while two subjects were relatives from each other (triangles). The outlier indicated by (*) used levodopa at the time of the study.

The SARA scores in patients varied between 3.5 and 15.5 with a significant correlation between these scores and the SCA3-RP LOOCV z-scores (ρ = 0.75, *P* = 0.001; Table 1 and Fig. 3). When SARA sub-scores were evaluated, SCA3-RP LOOCV expression z-scores correlated significantly with gait alteration (ρ = 0.59, *P* = 0.013) and disequilibrium at stance (ρ = 0.70, *P* = 0.002), but not with the other sub-scores (sitting instability, speech disturbance, limb dysmetria and dysdiadochokinesia). At alpha = 0.005 SCA3-RP LOOCV z-scores were not significantly correlated to neuropsychological test scores (Table 1). At the uncorrected significance level (*P* < 0.05), a moderate association was seen between letter fluency and SCA3-RP LOOCV z-scores (ρ = −0.51, *P* = 0.04), while letter fluency was also the only neuropsychological parameter that was significantly correlated to the (clinical) SARA scores (ρ = −0.618, *P* = 0.008).

### 3.5 Relation with grey matter atrophy

**Fig. 4:**
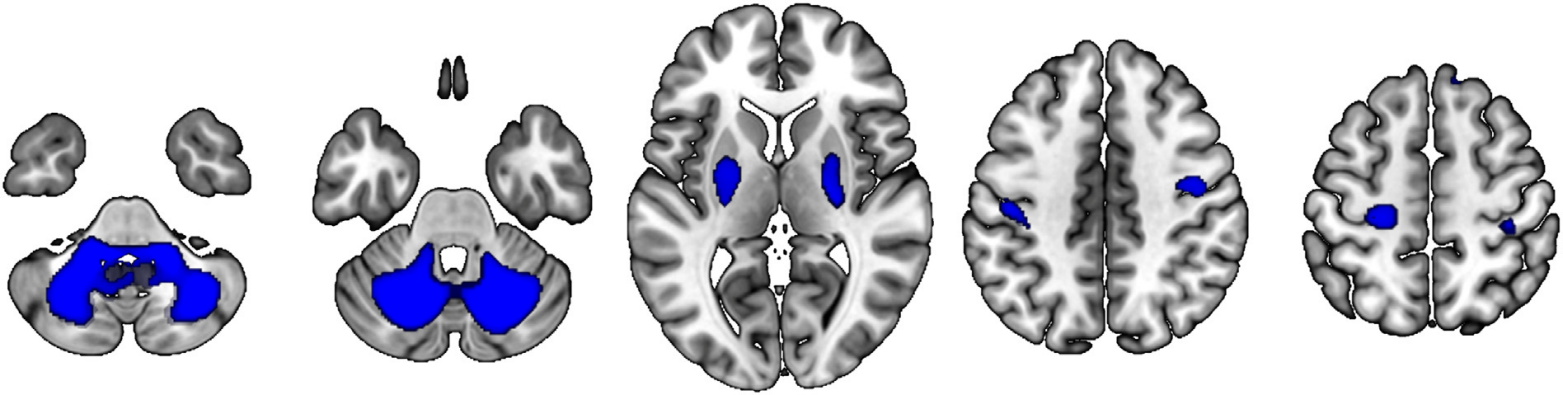
Brain regions showing grey matter atrophy in SCA3 compared to controls (*P* < 0.01, cluster extent 50). Only the cerebellar clusters are significant at a cluster defining threshold of *P* < 0.001, with cluster correction of *P* < 0.05.

VBM analysis revealed significant grey matter atrophy in SCA3 patients compared to controls in the cerebellum, bilaterally, including the right vermis (Table 2). At more lenient threshold (*P* < 0.01, uncorrected), regions with decreased grey matter volume included the striatum, bilaterally, and motor cortex (Fig. 4).

**Table 2:** VBM results (independent samples T-test, SCA3 < controls).

Region	Coordinates (MNI)			Cluster-extent	T-value (peak)	P-value (FWE cluster-corrected)
	x	y	z			
Cerebellum, L	−22.5	−64.5	−36	769	5.776251793	0.002
Cerebellum, R	19.5	−61.5	−33	821	5.176522255	0.002
Cerebellar vermis, R	10.5	−60	−27		4.56364584

**Fig. 5:**
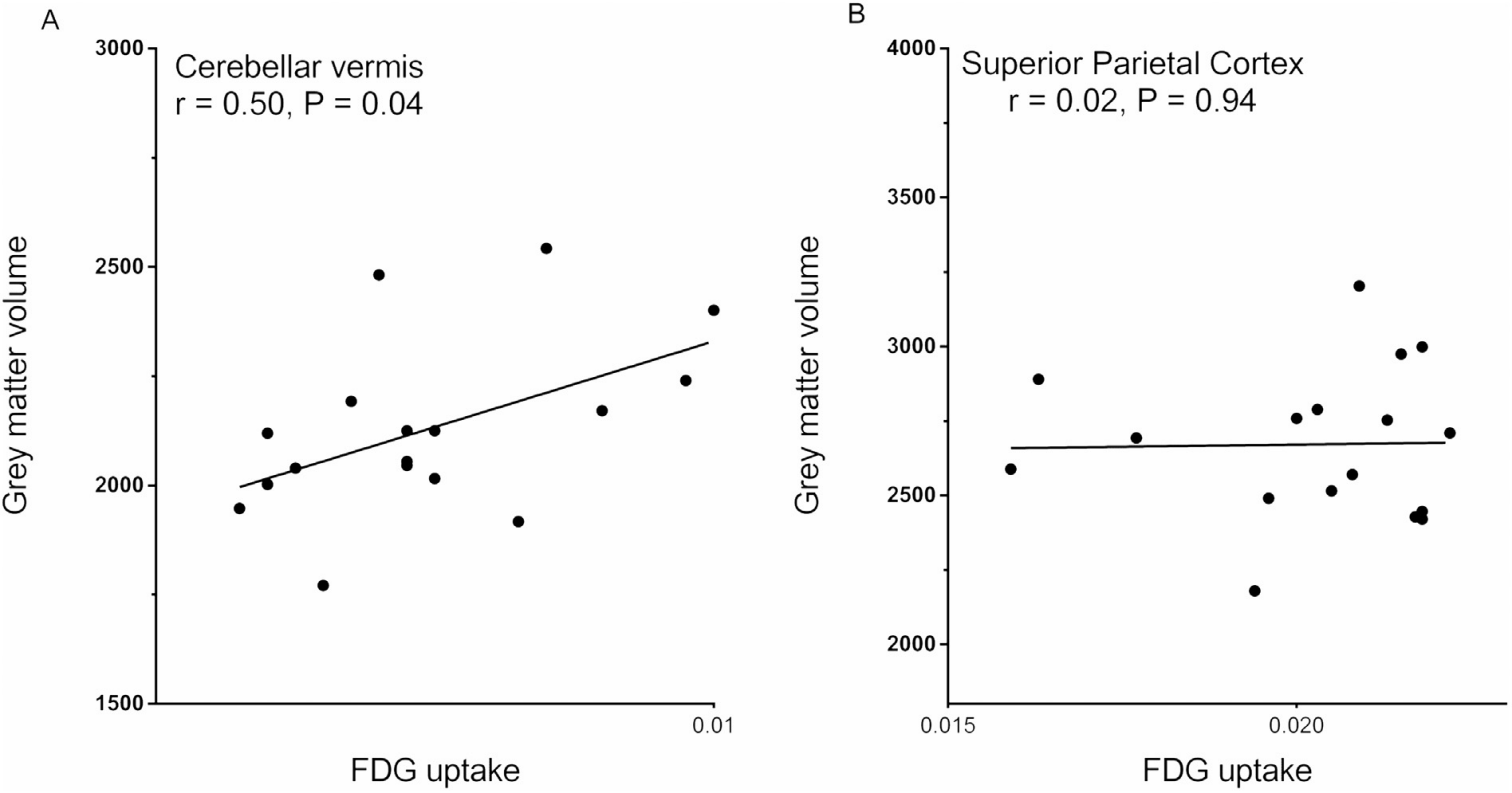
Correlation between grey matter volume and FDG uptake in the cerebellar vermis (A) and the superior parietal cortex (B).

The ROI analyses revealed a significant correlation between FDG uptake and grey matter volume in the cerebellar vermis (*r* = 0.50, P = 0.04; Fig. 5A), although this correlation did not survive correction for multiple comparisons. In the other ROIs, correlations were not significant (illustrated for the superior parietal cortex in Fig. 5B). SARA scores were correlated with grey matter volume in the caudate (ρ = −0.494; P = 0.04), and disease duration was correlated with FDG uptake in the thalamus (ρ = −0.584; *P* = 0.014). These correlations, however, were weak and did not survive correction for multiple comparisons.

## 4 Discussion

We identified a spatial metabolic pattern specific for SCA3 in a homogenous group of patients. The SCA3-RP was characterized by relative hypometabolism in the cerebellar hemispheres and vermis, the brainstem, caudate nucleus, and the posterior parietal cortex. Relatively increased metabolism was found in the cerebellar peduncles, the amygdala, hippocampus/parahippocampal gyrus, orbitofrontal cortex, insula, the anterior and middle cingulate gyrus and the somatosensory cortex.

Relative decreases in the metabolic SCA3-RP may reflect either impaired neuronal function due to cellular pathology at that location, or remote functional network changes due to lesions elsewhere. In the present study, some of the hypometabolic areas in the SCA3-RP do indeed overlap with areas known to be affected by pathology (Seidel et al., 2012; Rub et al., 2008; Rub et al., 2013): cerebellum, lower brainstem, midbrain, pallidum, thalamus, and primary motor cortex. However, the comparison of the anatomical MR images (T1) of our patients with the control MRI's, only revealed significant grey matter volume loss in the bilateral cerebellum and right cerebellar vermis, which is consistent with the morphological imaging literature (Hernandez-Castillo et al., 2017; Lukas et al., 2006; Reetz et al., 2013). Moreover, a (weak) correlation between FDG uptake and grey matter volume was only found in the cerebellar vermis, and not in other regions, which underscores that the hypometabolic pattern in the SCA3-RP does not simply reflect regional atrophy. As the cerebellum is the core region of pathology in SCA3, one might have expected a stronger correlation between grey matter volume and FDG uptake in the cerebellum. On the other hand, autopsy-studies have shown that neuronal loss in the cerebellar cortex is variable. In fact, Schöls et al. reported <25% Purkinje cell loss in most SCA3 cases (Schols et al., 2004). In addition, Shakkottai et al. demonstrated changes in Purkinje neuron firing that preceded the onset of Purkinje cell loss in a SCA3 mouse model (Shakkottai et al., 2011). This may provide arguments that the cerebellar component in the SCA3-RP reflects changes in neuronal activity that are not necessarily a consequence of only atrophy. The absence of significant correlations between FDG reduction and grey matter atrophy outside the cerebellum, further supports the involvement of remote functional changes.

Regions affected by neurodegenerative pathology may disrupt the integrity of distributed networks they participate in. This results in functional disconnection and dysfunction of spatially remote regions which are part of the same functional brain system. Due to widespread involvement of the cerebellum, brainstem, thalamus, pallidum, subthalamic nucleus (STN) and primary motor cortex, many functional brain systems are indeed affected in SCA3 (Rub et al., 2013). Focal disruption within the basal ganglia-thalamocortical loop may explain hypometabolism of the caudate nucleus and the putamen/pallidum. Moreover, previous studies have shown neuronal loss in the substantia nigra pars compacta, which was associated with decreased dopamine transporter binding in the putamen and caudate in SCA3 patients (Rub et al., 2008; Braga-Neto et al., 2012b; Yen et al., 2002). The combination of locally progressing pathology and the disruption of complex interacting motor networks could underlie the variability of the movement disorder seen in SCA3 (Pedroso et al., 2013).

Hypometabolism of the cerebellum, the posterior parietal cortex (left and right precuneus [BA7] and right inferior parietal lobule [BA39]) may reflect changes in coherent cerebellar-parietal functioning. As an example, one function to consider is feed-forward processing that is involved in predicting the (sensory) consequences of action (Blakemore and Sirigu, 2003; Ridderinkhof and Brass, 2015; Roth et al., 2013). A consequence of sub-optimal forward processing in (higher order) motor control may be an increased demand on actual movement correction, which is a core feature of ataxia. The latter may explain the relative increases we observed in the primary (Brodmann areas 3 and 2) and secondary (Brodmann area 5) somatosensory areas. Such enhanced anterior parietal activity may therefore represent a consequence of impaired forward processing in posterior parietal circuitry implicated in higher-order motor control.

Expression of the SCA3-RP correlated significantly with SARA scores. In contrast, SARA scores did not convincingly correlate to grey matter loss or FDG uptake in any single region. This supports the interpretation that the SCA3-RP represents network-level changes in neuronal activity implicated in the pathophysiology underlying ataxia in SCA3.

At the group-level, SCA3 patients showed impairments in letter fluency and processing speed and a moderate association was seen between letter fluency and SCA3-RP subject scores. The strong association between the SCA3-RP and SARA scores supports the notion that this pattern primarily reflects ataxia, a symptom that all SCA3 patients have in common, which is not the case for the more variable expression of cognitive and affective symptoms in the patient group.

Our findings of relative hypermetabolism in the limbic system, including the hippocampus, seem at odds with results of other functional imaging studies, which have reported hypometabolism (Wang et al., 2007) and hypoperfusion (Braga-Neto et al., 2012a) of the parahippocampal gyrus. In our study, hippocampal hyperactivity may reflect relative sparing of this area, but it may also reflect a compensatory mechanism. It is possible that the SCA3-RP changes with disease progression, and that the hippocampus becomes hypo-active in more advanced stages of the disease.

In conclusion, this study demonstrates that SCA3 patients exhibit a metabolic profile that not only includes cerebellum and brainstem, but also striatum and parietal cortex. The SCA3-RP and its correlation to clinical measures should be validated in a larger, novel cohort, preferably also in patients with a shorter disease duration or even in pre-clinical individuals. In addition, in order to determine the specificity of the pattern for SCA3, it would be of interest to compute SCA3-RP expression in FDG-PET scans of patients with other cerebellar ataxias. Longitudinal imaging may help understand the metabolic changes with disease progression, and their relation to clinical features such as cognitive decline. Metabolic patterns may help to predict disease onset in pre-clinical individuals (Tang et al., 2013). Such imaging biomarkers may become particularly important in the future, as new therapeutic interventions are designed with the aim of disease modification.

## Author's roles

Sanne K. Meles: analysis and interpretation of data, drafting of the manuscript.

Jelmer G. Kok: acquisition of data, analysis and interpretation of data, drafting of the manuscript.

Bauke M. De Jong: study concept and design, interpretation of data, drafting the manuscript, study supervision, critical revision of manuscript for intellectual content.

Remco J. Renken: supervision of imaging data analysis, critical revision of manuscript for intellectual content.

Jeroen J. de Vries: acquisition of data, critical revision of manuscript for intellectual content.

Jacoba M. Spikman: supervision of neuropsychology data analysis, critical revision of manuscript for intellectual content.

Aaltje L. Ziengs: acquisition of neuropsychology data.

Antoon T.M. Willemsen: acquisition of data, critical revision of manuscript for intellectual content.

Harm J. van der Horn: analysis and interpretation of data, critical revision of manuscript for intellectual content.

Klaus L. Leenders: critical revision of manuscript for intellectual content.

Hubertus P. H. Kremer: study concept and design, interpretation of data, critical revision of manuscript for intellectual content, study supervision.

## Financial disclosure/conflict of interest

The authors report no conflicts of interest.

## Funding

SKM was funded by a grant from the Dutch “Stichting ParkinsonFonds”. JGK was funded by a grant from the U4 network.

## Appendix A Supplementary data

Supplementary figures Image 1
