# Supplementary material for: The cerebral metabolic topography of spinocerebellar ataxia type 3
Source: Neuroimage Clin. 2018 Mar 29;19:90–7. doi: 10.1016/j.nicl.2018.03.038 (PMC6051313; doi:10.1016/j.nicl.2018.03.038)
Supplement: Supplementary file 1 — Supplementary figures [file mmc1.docx]

Supplementary Figure 1. Correlation between CAG repeat length and age at onset





Supplementary Figure 2. Unthresholded original SCA3-RP


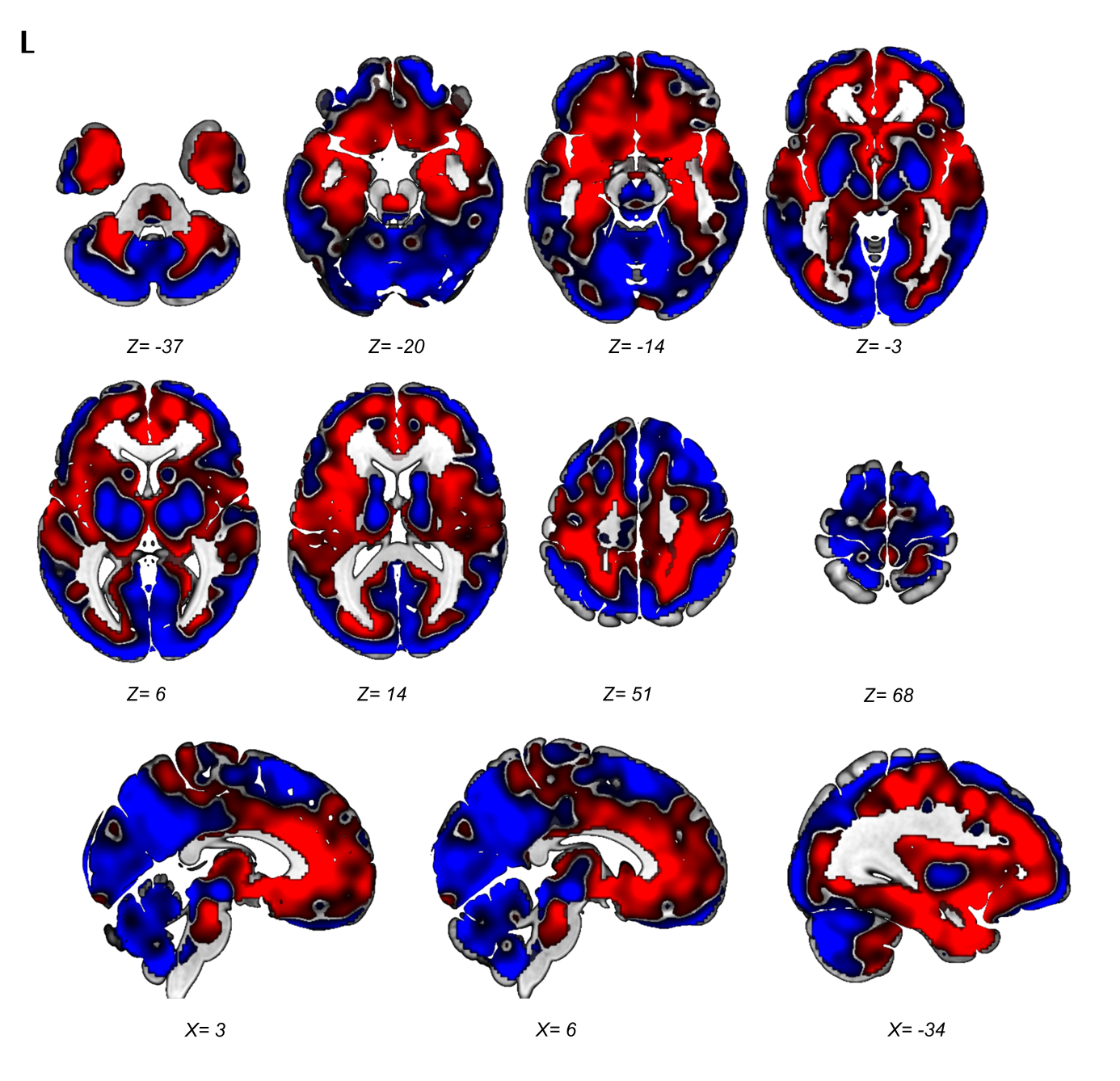


The original, unthresholded SCA3-RP overlaid on a T1 MRI template. Red indicates positive voxel weights (relative hypermetabolism) and blue indicates negative voxel weights (relative hypometabolism). L=left. Coordinates in the axial (Z) and sagittal (X) planes are in Montreal Neurological Institute (MNI) standard space.

Supplementary Figure 3. Mean profile for controls and patients


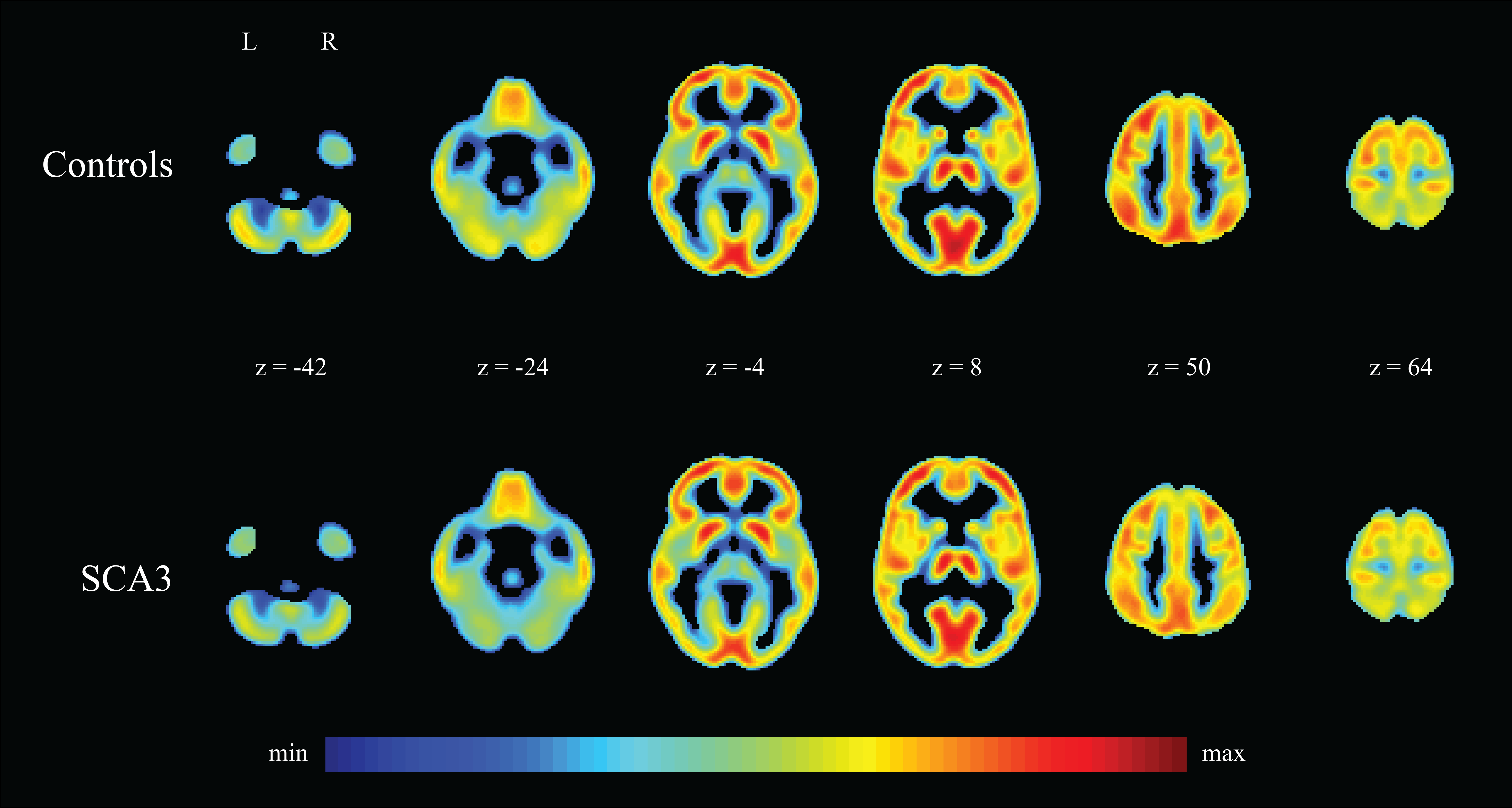


After registration and masking, raw count data were log-transformed and the mean of each subject was removed for normalization purposes. Subsequently, the mean profile for patients and controls separately was determined and displayed in this figure to give an impression of average FDG distribution in both groups.
